# Measuring the impact of dietary supplementation with citrus or cucumber extract on chicken gut microbiota using 16s rRNA gene sequencing

**DOI:** 10.1007/s11259-024-10417-w

**Published:** 2024-05-23

**Authors:** Francesca Riva, David H. McGuinness, Dorothy E. F. McKeegan, Jorge Peinado-Izaguerri, Geert Bruggeman, David Hermans, Peter D. Eckersall, Mark McLaughlin, Maureen Bain

**Affiliations:** 1 School of Health and Life Sciences, University of the West of Scotland, High St, PA1 2BE Paisley, UK; 2 School of Biodiversity, One Health and Veterinary Medicine, University of Glasgow, Bearsden Rd, G61 1QH Glasgow, UK; 3 Faculty of Veterinary Medicine, University of Zagreb, Radoslava Cimermana, 10000 Zagreb, Croatia; 4 Glasgow Polyomics, University of Glasgow, Switchback Rd, G61 1BD Bearsden, Glasgow, UK; 5 Department of Microbiology and Immunology, University of Veterinary Medicine and Pharmacy in Ko?ice, Komensk?ho, 041 81 Ko?ice, Slovakia; 6 School of Biological Sciences, The University of Manchester, Oxford Rd, M13 9PT Manchester, UK; 7 Nutrition Sciences N. V, B-9031 Booiebos, Ghent, Belgium

**Keywords:** Broiler chicken, Novel plant extracts, Gut microbiota, 16SrRNA gene sequencing, Citrus, Cucumber

## Abstract

This study investigated the effects of dietary supplements, citrus (CTS) and cucumber (CMB), on the jejunum and cecum microbiota of 14- and 28-days old broiler chickens to evaluate their impact on the gut health and assess their role as alternatives to antibiotic growth promoters (ABGPs). 16SrRNA gene sequencing revealed the overall bacterial microbiota composition was significantly affected by the gut site (*p*?<?0.001) but not by either of the dietary supplements, CTS and CMB, at both 14 and 28 days of age. However, as a result of Linear discriminant analysis (LDA) effect size (LEfSE), CTS dietary supplements significantly increased the counts of *Lactobacillus* (*p*?<?0.01) and decreased the counts of *Enterococcus* (*p*?<?0.01) and *Clostridium* (*p*?<?0.05) in the jejunum, whereas the counts of *Blautia* were increased (*p*?<?0.01) and *Enterococcus* were decreased (*p*?<?0.05) in the cecum at both ages. Only minor CMB effects were identified in the cecum and non in the jejunum. The use of CTS dietary supplements has been shown to be associated to the reduction of potentially pathogenic bacteria (*Enterococcus* and *Clostridium*) and to the growth of beneficial bacteria (*Lactobacillus* and *Blautia*) which are known to have positive effects on chicken health in terms of nutrients absorption, stimulation and production of short chain fatty acids (SCFAs). Therefore, this study suggests that the use of a CTS supplemented diet could promote gut health while no clear advantages have been identified with the use of CMB as a dietary supplement.

**Supplementary Information:**

The online version contains supplementary material available at 10.1007/s11259-024-10417-w.

## Introduction

For many years antimicrobial growth promoters (ABGPs) have been routinely used in broiler chicken diets to promote their growth and prevent the onset of diseases (Moore at al. 1946; Mehdi et al. 2018). Since 2006, ABGPs have been banned from poultry feed in EU livestock, as their use was associated with the development of antimicrobial resistance (AMR) (Moore and Evenson 1946; Mehdi et al. 2018; Vanderhaeghen and Dewulf 2017). The withdrawal of ABGPs may lead to increased bird disease rate and consequently a rise in the use of antibiotics for therapeutic scope (Casewell et al. 2003). Thus, there is a need to find alternatives that improve broilers health while maintaining production efficiency and product safety (Mehdi et al. 2018; Ayalew et al. 2022). Although the mechanism of action that AGPs uses to enhance animal performance is still unclear, it is believed that they act mainly through modulation of the gastrointestinal microbiota (Dibner and Richards 2005). The diversity and complexity of gut microbiota in broilers can be influenced by the diet, gut region, age and environmental factors (Feye et al. 2020). Novel plant extracts, such as citrus (CTS) and cucumber (CMB) extracts, have been identified as viable alternatives in broiler nutrition due to the potential beneficial effect associated to their bioactive compounds, wide availability and a cost effective purification process (Savoia 2012; Chen et al. 2006; Kamboh et al. 2016; Csernus et al. 2020). CTS extracts are particularly rich in pectin (a source of soluble fiber), polyphenols (including flavonoids), carotenoids, and essential oils (including limonene) (Rafiq et al. 2018). Dietary fibers such as pectin cannot be digested and absorbed by the small intestine, but instead they undergo microbial fermentation by commensal gut bacteria leading to the production of various metabolites, most importantly the short-chain fatty acids (SCFA) (Sahasrabudhe et al. 2018). Many studies have demonstrated that SCFAs play a significant role in the regulation of the gut health of poultry (Liu et al. 2021b) by improving the immune system, inhibiting intestinal inflammation and regulating the gut environment (Cani 2014). Polyphenols, carotenoids, and limonene have been shown to have antioxidant and anti-inflammatory properties (Prihambodo et al. 2021; Mavrommatis et al. 2022; Agatemor et al. 2015). Likewise, CMB extract contains vitamins, ?-carotene and polyphenols, making it a great candidate for the modulation of the broiler microbial population and immune system (Tang et al. 2010; Vouldoukis et al. 2004; Bernardini et al. 2018). Accumulating evidence has suggested that both dietary supplements, CTS and CMB, could positively modulate the broiler chicken gut microbiota by promoting the growth of beneficial bacteria. However, data from other studies suggest that the overall chicken gut microbiota is more likely to be influenced by the gut site rather than dietary supplements (Ballou et al. 2016). Each region of the broiler?s gut can be differentiated both morphologically and functionally: the chicken foregut (duodenum, jejunum, ileum) is mainly responsible for the digestion and absorption of nutrients, while the hindgut (cecum, colorectum) is the main site of microbial fermentation (Glendinning and Watson 2019). Studies on the influence of diets on the microbiota in broilers have mainly focused on microbes in the ileum and cecum as they showed the most diverse bacterial populations, whereas few studies have focused on those in the duodenum and jejunum (Glendinning and Watson 2019; Haghighi et al. 2006).

Microbiota diversity increases along with the development and growth of chickens until it becomes a relatively stable microbiota composition (Crhanova et al. 2011). In broilers, Huang et al. 2018 showed that the bacterial diversity peaked at 14 days of age in the small intestine and 28 days of age in the large intestine. However, Lu et al. 2003 demonstrated that the broiler?s cecal microbial community resulted in no differences between 14 and 28 days of age.

Accordingly, our research approach was to evaluate the effect of CTS and CMB diets on the jejunum and cecum microbiota of 14- and 28-day-old broiler chickens as well as characterize the microbial diversity between tissues. The hypothesis being investigated was that the active compounds of the CTS or CMB diets would modulate the host microbiota and stimulate the growth of beneficial bacteria and that these might indicate that such additives would prove of value as an alternative to ABGPs.

## Materials and methods

### In vivo trial and sampling

A total of 108?day-old male broiler chickens (Ross 308) were collected from a commercial hatchery (PD Hook Hatcheries Ltd, Bampton, UK) and reared for 28 days at Cochno Farm and Research Centre, Glasgow. At day 0, each bird was individually wing-tagged, weighed and randomly allotted to three dietary treatments: starter diet without any supplements (CTL), starter diet with citrus extract supplement (CTS) (300?mg/kg), starter diet with cucumber extract supplement (CMB) (75?mg/kg). The chicken starter (day 0 to 14) and grower (day 14 to 28) corn-soybean meal-based diets were formulated and prepared at NuScience in Ghent, Belgium (Table?1). Each experimental dietary group consisted of 9 chickens randomly allocated to each of the 12 pens (4 replicate pens/ diet) of 2.5 m^2^ size on a litter of wood shavings. Each pen was equipped with with a spot brooder, feeder, drinker and woodshavings litter. Broilers were provided ad libitum access to water and supplied feed throughout the experiment. The lighting and heating regime followed the recommendations of the Ross 308 breeder management guide (Aviagen, Midlothian, UK); lighting started with 23-h light and 1-h darkness (23?L:1D) from day 0 to day 7 and gradually decreased to 18?L:6D on day 28. The room temperature was set at 35??C at the start of the experiment and gradually decreased of 1??C each three days until 20??C at day 28 with humidity?>?50%.

**Table 1:** Feed ingredients (g/100kg) of basal starter and grower diet for broiler chickens from 0 to 28 days of age

Feed ingredients	Starter diet (g/100kg) (0 to 14 days)	Grower diet (g/100kg) (14 to 28 days)
Corn	25,000	25,000
DL-methionine	0,107	0,081
L-Lysine HCI	0,234	0,266
L-Threonine	0,097	0,105
Premix Minevita Bro	3,000	3,000
Monteban 100	0,060	0,060
Sodium Bicarbonate	0,196	0,089
Soya bean meal 47%CP?+?2%CP	27,450	22,114
Soya bean oil refined	1,880	3,072
Soya beans Danex	7,500	7,500
Vit Choline Chloride 60% Veg.	0,010	0,010
Xylanase	0,010	0,010
Wheat enzymes	34,456	38,636
Monocalcium Phosphate	0,000	0,031
Salt	0,000	0,025

### Sample collection and DNA extraction

At 14, 21 and 28 days old, 12 broilers per diet were randomly selected, weighed (see Supplementary File 1) and humanely euthanised by anaesthetic overdose (1?ml/kg of Pentobarbital sodium R Euthatal Dopharma Research B.V.), injected into the brachial vein. Only chickens euthanised at day 14 and 28 were used for the microbiota investigation (Table?2). The sex of each bird was determined by dissection (33 males and 3 females at 14 days old, 34 males and 2 females at 28 days old) and the females were excluded from the study to maintain consistency. The mucosa and digesta of the proximal jejunum (section of 5?cm), and one cecum (entire length) were collected from each bird by gentle scraping with a glass slide. The genomic DNA was extracted from ~?250?mg of jejunum and ceca samples following the DNeasy PowerSoil Pro kit (Qiagen, Manchester, UK) protocol. The DNA concentration and purity were controlled by the High Sensitivity DNA Qubit system (ThermoFisher, Paisley, UK). The DNA concentration, ng/?l, was assessed at 260?nm while the ratio of absorbance at 260 and 280?nm was used to assess DNA purity.

**Table 2:** Experimental design. At day 0, a total of 108 male broiler chickens were raised in 12 pens (4 replicate pens per each diet). 9 chickens were allocated to each pen. At day 14 and 28, 3 chickens per pen were randomly sampled and gut tissues (jejunum and cecum) collected. The ramaining birds were used for a separate study

	Day 0 (total number of birds)			Day 14 (number of birds collected)			Day 28 (number of birds collected)		
Diet Pen	CTL	CTS	CMB	CTL	CTS	CMB	CTL	CTS	CMB
A1-A4	9	//	//	3	//	//	3	//	//
B1-B4	//	9	//	//	3	//	//	3	//
C1-C4	//	//	9	//	//	3	//	//	3
Total	36	36	36	12	12	12	12	12	12
	108			36			36		

### 16S library preparation and sequencing

The 16S library preparation and sequencing was performed using the Illumina (San Diego, CA) protocol and Nextera XT DNA Library Prep Kit (Illumina, San Diego, CA) for the library preparation workflow. The V3-V4 region of the 16S ribosomal RNA (rRNA) gene was amplified with a specific 2-step PCR. During the first PCR reaction, 12.5 ng DNA per each sample, the Illumina Forward Primer 5?-CTTACGGGNGGCWGCAG- 3?, Reverse Primer 5? -GACTACHVGGGTATCTAATCC- 3?, and the 16 unique nucleotide-barcodes associated to each sample, were used. The Illumina primers were designed to have an overlap sequence to make them compatible with Nextera identifier indices and sequencing adaptors which were attached during the second PCR step. PCR reactions were performed by initial denaturation at 95??C for 3?min and then 25 cycles at 95??C (30?s), 55??C (30?s), 72??C (30?s) and the final elongation step at 72??C for 5?min. The amplicons were quantified by the High Sensitivity DNA Qubit system (ThermoFisher, Paisley, UK) and sequenced using the Illumina MiSeq platform (Illumina, San Diego, CA, USA).

### Bioinformatics and statistical analysis

Sequence reads were processed using the Quantitative Insights Into Microbial Ecology (QIIME?) v1.9.0 bioinformatics pipeline (Carporaso et al. 2010). Briefly, sequences were demultiplexed and filtered according to the read length threshold (250?bp). Barcodes and primer sequences were trimmed using Cutadapt v1.18 (Martin 2011) and paired-end reads were merged into single assembled reads using *Pandaseq* v2.10 (Masella et al. 2012). Low quality sequences (Phred quality score?<?25, read length?<?250?bp) were excluded. No ambiguous, or ?N? calls were present in the data as assessed by FastQC (Andrews 2010). The chimeric sequences were also identified and removed. The remaining quality-filtered reads were clustered into operational taxonomic units (OTUs) (97% similarity threshold) and sequences from each OTU referenced against the Greengenes database (v13_5) using the PyNAST method, to assign taxonomy (Caporaso et al. 2010). A Biological Observation Matrix (BIOM) table was generated, low abundance sequences (<?0.005% relative abundance) were removed (Bokulich et al. 2013), and the table was rarefied to 5,000 sequences per sample. ?-diversity and ?-diversity analyses were performed on the rarefied OTU tables to assess the microbial community diversities based on different dietary administration (CTL, CTS, CMB) and gut site (jejunum and cecum). Data of broilers at 14 and 28 days old were combined to increase the sample size as only subtle differences were discovered based on bird age (see Supplementary File 2). The ?-diversity indexes, Chao1 and PD_whole_tree, were used to evaluate the species richness and phylogenetic diversity. To compare the microbial similarity between individual samples, the unweighted UniFrac distance metrices were computed using the OTU table and phylogenetic tree information to generate the Principal coordinate analysis (PCoA) plots. Group comparisons were corrected using the Benjamin-Hochberg False Discovery Rate (FDR) procedure. The unweighted UniFrac distance metrices was used as the sensitive and qualitative measure to ensure that low abundance features were not obscured. The two-dimensional PCoA were generated in RStudio (version 4.0.0) using the Unweighted UniFrac distance matrix from QIIME?. The nonparametric Permutational Multivariate Analysis of Variance (PERMANOVA) tests were used to statistically determined differences between the dietary and tissue groups explained by the PCoA. In addition, the Linear discriminant analysis (LDA) effect size (LEfSE) package (Segata et al. 2011) was used to identify the differentially abundant taxa per each diet and tissue. The LEfSe algorithm uses the nonparametric factorial Kruskal- Wallis test (??=?0.05) to analyse differences between classes (i.e. diet) and the pairwise Wilcoxon test (??=?0.05) to check differences among subclasses (i.e. tissue). A bar chart representing the effect size (LDA) was produced, and a cladogram was generated to provide a visual representation of the phylogenetic tree. LDA scores (log10) greater than +/-2 indicate a statistically significant difference between the groups (*p*?<?0.05). The relative abundance of each significant bacterial strain was studied in each sample to determine its consistency within samples of the same group.

## Results

### Sequence analysis and quality filtering

A total of 3,189,407 sequencing reads were obtained from the jejunal and caecal samples. After removing low quality and chimeric sequences, the average number of reads generated per chicken was 23,327 for jejunal samples and 23,783 for caecal samples. In total, 4760 OTUs were identified at a 97% sequence similarity level with high threshold identity. After rare OTUs (<?0.005% of total OTUs) were filtered out, an average of 370 OTUs in the cecum and 270 OTUs in the jejunum for each sample were retained for the analyses.

### Effect of CTS and CMB on the jejunum microbiota

Rarefaction curves generated from the within community ?-diversity of CTL, CTS and CMB diets showed that no dietary effects were observed based on the total number of species in a sample (species richness) and phylogenetic diversity using the Chao1 and PD whole tree indexes (*p*?>?0.05) (Fig.?1a and b). Each rarefaction curve follows the same trend and approaches a plateau indicating that the sequencing depth (>?5000 sequences per sample) was sufficient to identify all OTUs accurately. Permutational Multivariate Analysis of Variance (PERMANOVA) tests showed that ?-diversity metrics between groups was not statistically different (*p*?>?0.05). No clustering of dietary treatments was identified by the Unweighted UniFrac distance Principal Coordinate Analysis (PCoA) plot (Fig.?1c).

**Fig. 1:**
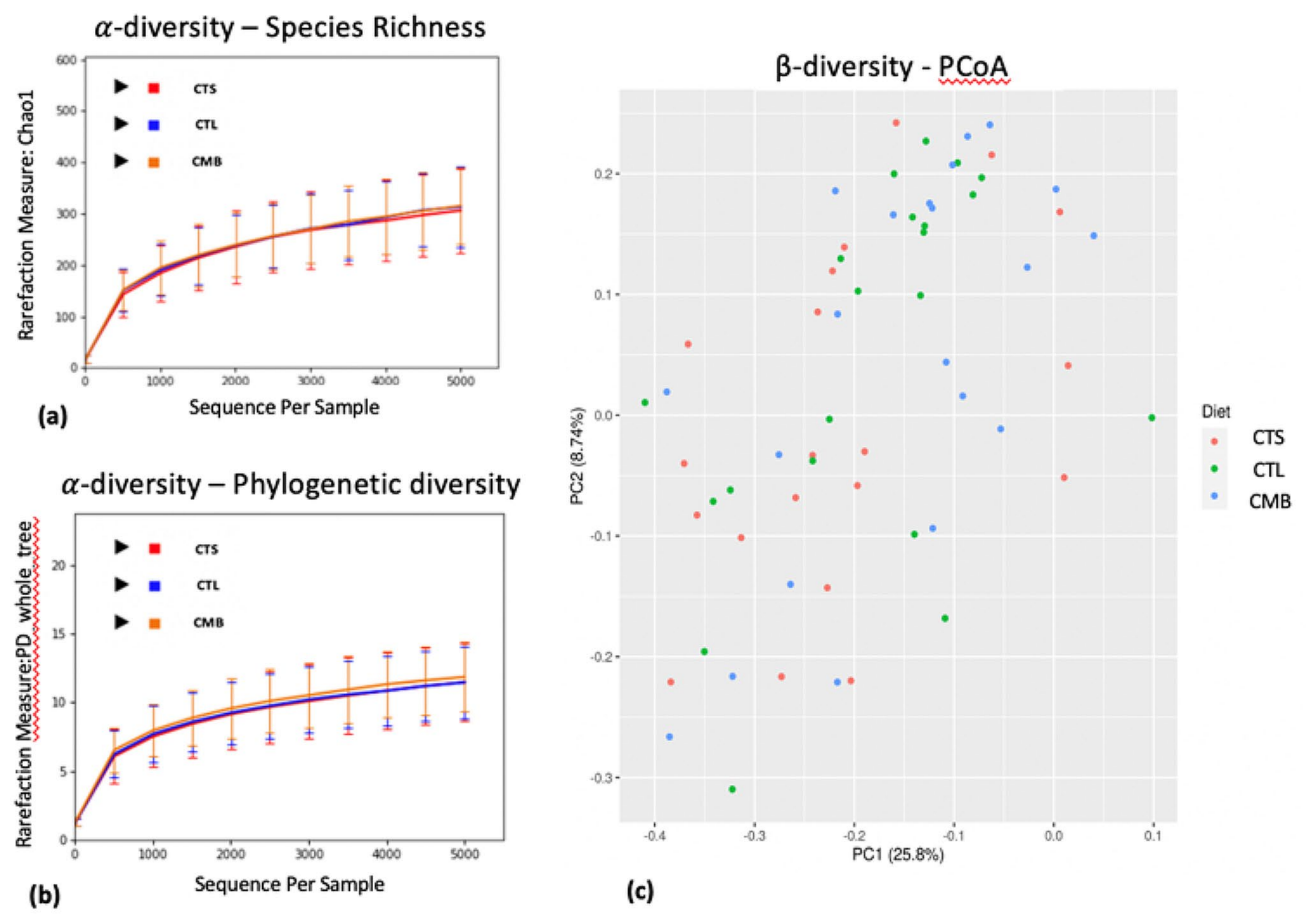
Dietary effect on microbiota diversity in the jejunum. The three rarefaction curves calculated at the lowest subsample size of 5000 sequences per sample indicate the effect of sequencing on the species richness (Chao1) (**a**) and phylogenetic diversity (PD_whole_tree) (**b**). No differences within a given sample, depending on diets (CTL vs. CTS vs. CMB) are shown in terms of both indices. Principal Coordinate Analysis (PCoA) of unweighted UniFrac distances shows no clustering among samples associated with a given diet. PC1 explained 25.8% of variation and PC2 explained 8.74% of variation (**c**). CTL: diet without additives, CTS: CTL diet supplemented with citrus extract (300?mg/kg diet), CMB: CTL diet supplmented with cucumber extract (75?mg/kg diet)

LEfSE results showed 21 taxonomic biomarkers in the jejunum of broilers fed a CTS diet when compared to the CTL diet. LEfSE identified a marked increase of the *Lactobacillus* genus and *Lactobacillaceae* family in the CTS diet (LDA?>???4). The relative abundance of other genus such as *Streptomyces, Rhodocuccus* and the families *Streptomycetaceae, Nocardiaceae* was also found significantly higher in the CTS group compared to the CTL dietary group (LDA?>???2). A marked decrease of the genus *Enterococcus* and the family *Enterococcaceae* was identified in broilers fed the CTS diet (LDA?>?4). Similarly, the *Clostridium* genus and families of *Aerococcaceae* and *Clostridiaceae* showed a lower abundance in CTS diet when compared to the CTL group (LDA?>?2) (Fig.?2a). The relative abundance of the 21 bacteria was studied in each sample; the increase abundance of *Lactobacillus* and decrease abundance of *Enterococcus* and *Clostridium* was confirmed in the jejunum of broilers fed with CTS diet (Fig.?2b, c and d). No differences were found in the jejunum using the CMB diet.

**Fig. 2:**
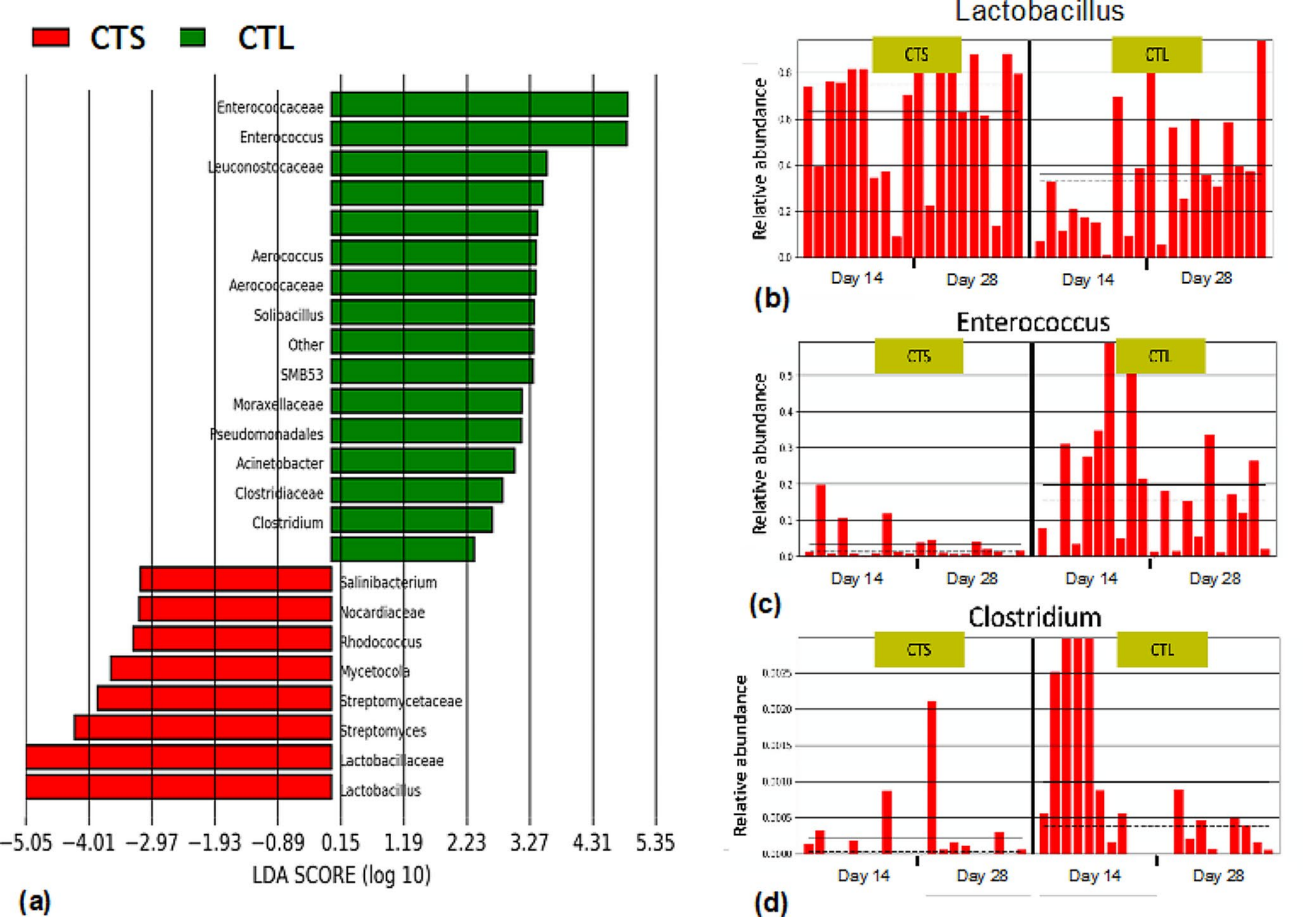
Bacterial strains modulated by CTS diet in the jejunum. Linear discriminant analysis (LDA) effect size (LEfSe) was used to identify specific phylotypes which are significantly influenced by CTS diet compared to CTL basal diet (*p*?<?0.05). A negative LDA score indicates the depletion of those bacteria in CTL diet and enrichment in CTS diet (red) while a positive LDA score represents the opposite. The LDA scores (log10) threshold +/- 2 indicates a statistically significant difference between the groups (*p*?<?0.05); a higher +/- LDA score indicate a bigger difference in the abundance of that bacteria in the specific dietary group (**a**). The three histograms indicate the relative abundance of the genus *Lactobacillus* (**b**), *Enterococcus* (**c**) and *Clostridium* (**d**). Each bar indicates the relative abundance of the taxa in each sample of the CTS and CTL diets at 14 and 28 days of age. CTL: diet without additives, CTS: CTL diet supplemented with citrus extract (300?mg/kg diet)

### Effect of CTS and CMB on the cecum microbiota

Rarefaction curves generated from both ?- diversity indexes, Chao1 and PD_whole_tree, indicate that diets (CTL vs. CTS cs CMB) did not statistically differ in terms of within sample bacterial diversity (*p*?>?0.05) (Fig.?3a and b). Permutational Multivariate Analysis of Variance (PERMANOVA) tests confirmed that ?-diversity metrics between the groups was not statistically different (*p*?>?0.05). The Principal Coordinate analysis (PCoA) plot which was generated using the Unweighted UniFrac distance matrix demonstrated that cecum samples did not differ in terms of dietary treatment (Fig.?3c).

**Fig. 3:**
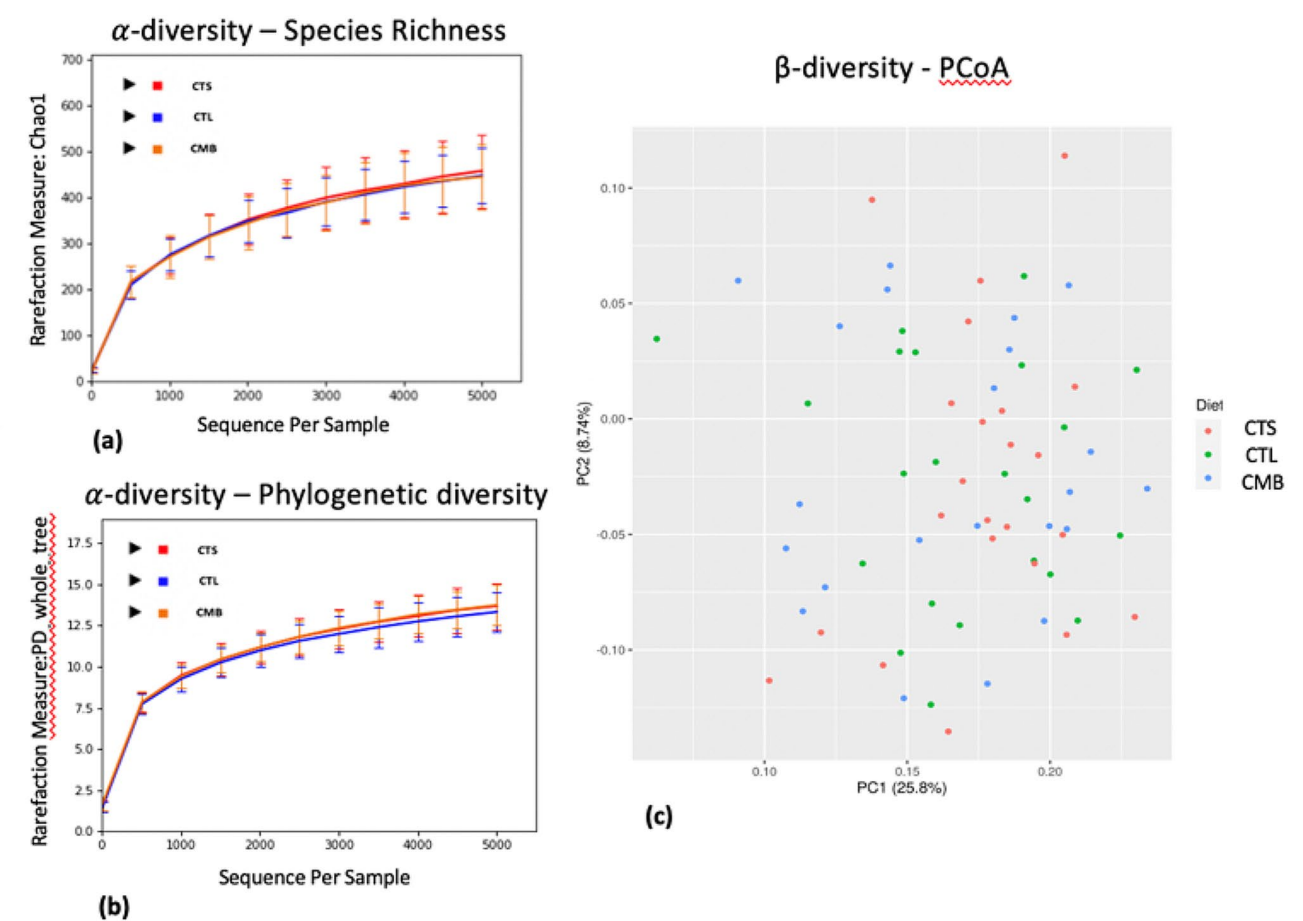
Dietary effect on microbiota diversity in the cecum. ?-diversity rarefaction curves, calculated at the size of 5000 sequences per samples, indicate that there are no differences in terms of species richness (Chao1) (**a**) and phylogenetic diversity (PD_whole_tree) (**b**) within a given sample depending on experimental diets (CTS and CMB). ?-diversity demonstrates through Principal Coordinate Analysis (PCoA) of unweighted UniFrac distances, that diets (CTS and CMB) do not modulate the overall microbiome composition. No clustering is formed based on the diets (**c**). CTL: diet without additives, CTS: CTL diet supplemented with citrus extract (300?g/t diet), CMB: CTL diet supplmented with cucumber extract (75?mg/kg diet)

Differentially abundant taxa between the dietary treatments were identified using LEfSe analysis (Fig.?4a). The comparison of CTS vs. CTL diet mainly revealed that the abundance of the genus *Blautia* (LDA?>???4) and *Enterococcus* (LDA?>?3) were markedly increased and decreased respectively in the CTS dietary group. Figure?4b and c show the histogram of the relative abundance of *Blautia* and *Enterococcus* in broilers fed the CTS and CTL diets at 14 and 28 days of age.

**Fig. 4:**
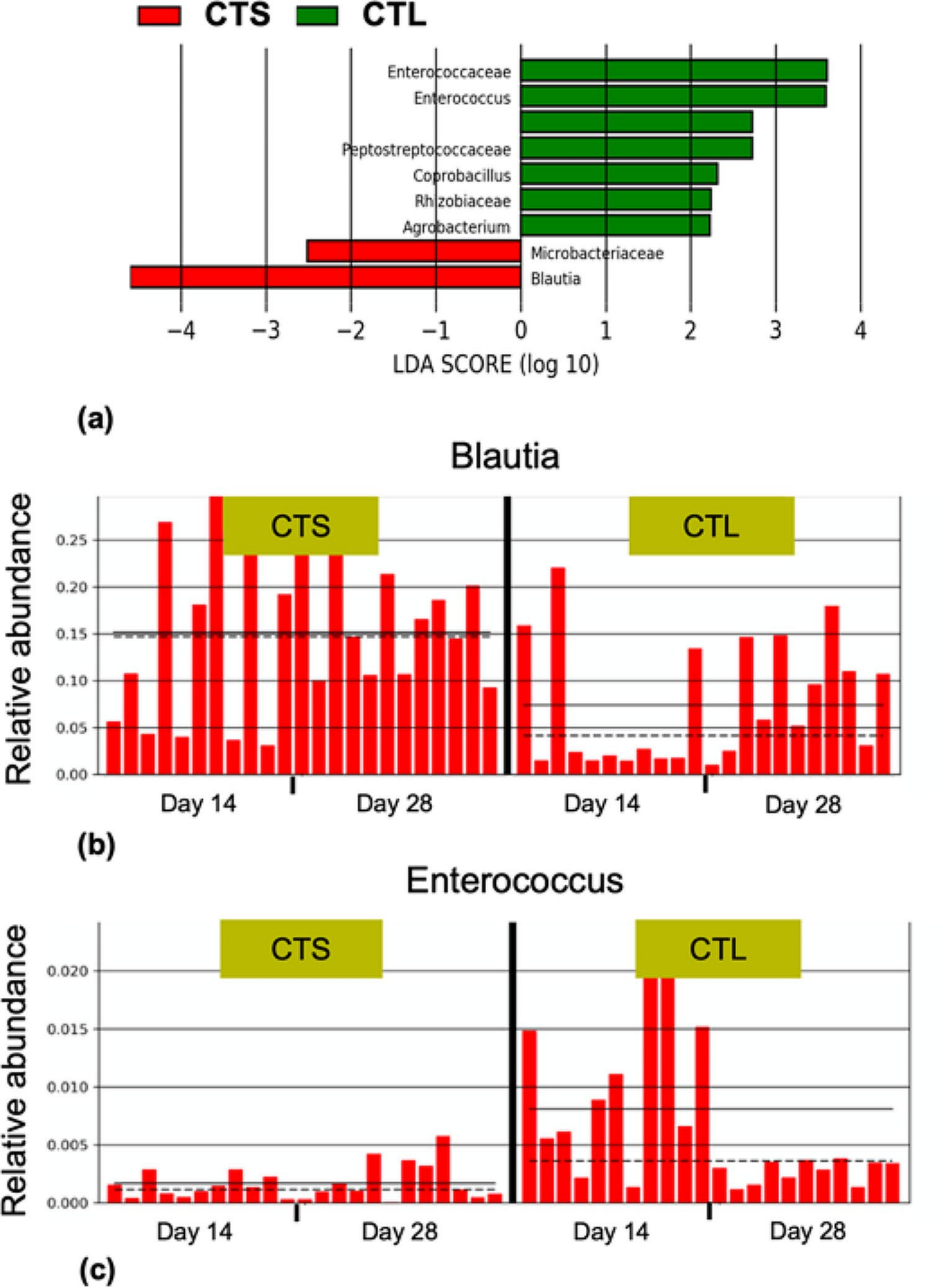
Bacterial strains modulated by CTS in the cecum. Linear discriminant analysis (LDA) effect size (LEfSe) identified specific phylotypes which were significantly influenced by CTS and CTL diets (*p*?<?0.05). The LDA scores (log10) threshold +/- 2 indicates a statistically significant difference between the two groups (*p*?<?0.05) (**a**). The two histograms indicate the relative abundance of *Blautia* (**b**) and *Enterococcus *(**c**) in each sample of CTS and CTL diets at 14 and 28 days. CTL: diet without additives, CTS: CTL diet supplemented with citrus extract (300?mg/kg diet)

CMB was also found to modulate 13 taxonomic bacterial strains in the cecum of broilers (Fig.?5a). Among these, only *Bacillus* (LDA?>?2) and the respective phylum and family (LDA?>?3) were confirmed to be influenced by CMB diet when compared to the CTL dietary group (Fig.?5b).

**Fig. 5:**
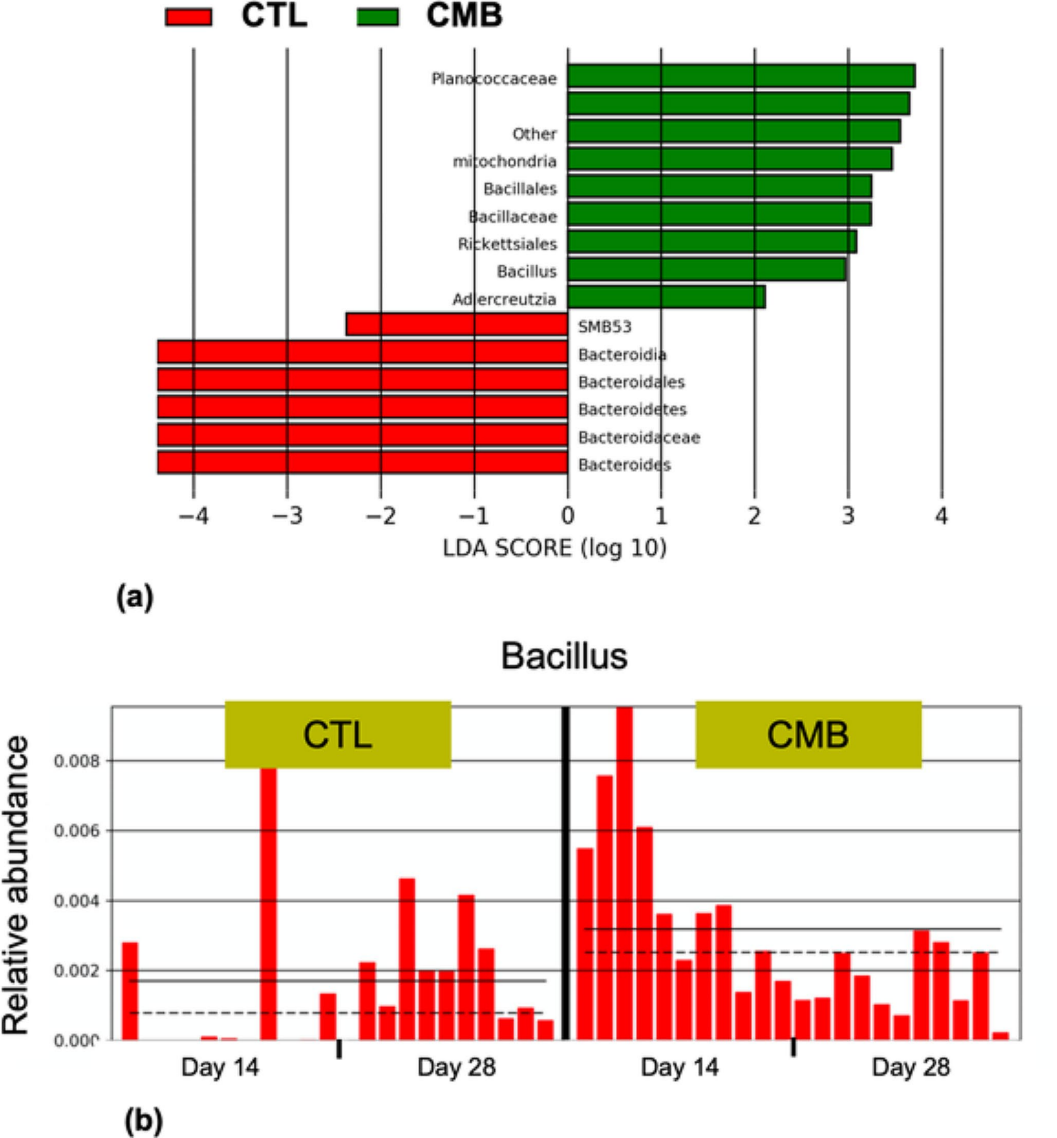
Bacterial strains modulated by CMB in the cecum. Linear discriminant analysis (LDA) effect size (LEfSe) indicates, through LDA scores (log10), the difference in abundance of specific bacteria belonging to CTL vs. CMB groups (*p*?<?0.05). The threshold LDA scores (log10) +/- 2 is used to identify the statistically significant difference between the groups (*p*?<?0.05) (**a**). The histogram (**b**) shows the relative abundance of *Bacillus* in broilers fed CMB and CTL diets at 14 and 28 days. CTL: diet without additives, CMB: CTL diet supplmented with cucumber extract (75?mg/kg diet)

### Comparison between jejunum and cecum microbiota

The two clearly separating rarefaction curves generated by Chao1 (Fig.?6a) and PD_whole_tree (Fig.?6c) indexes confirm that the gut site significantly affected the species richness and phylogenetic diversity of the microbiota (*p*?=?0.001). The Permutational Multivariate Analysis of Variance (PERMANOVA) has shown that ?-diversity metrics were statistically different between tissues (*p*?=?0.001). Principal Coordinate Analysis (PCoA) plots were generated using ?-diversity metrics and are presented in Fig.?6c.

**Fig. 6:**
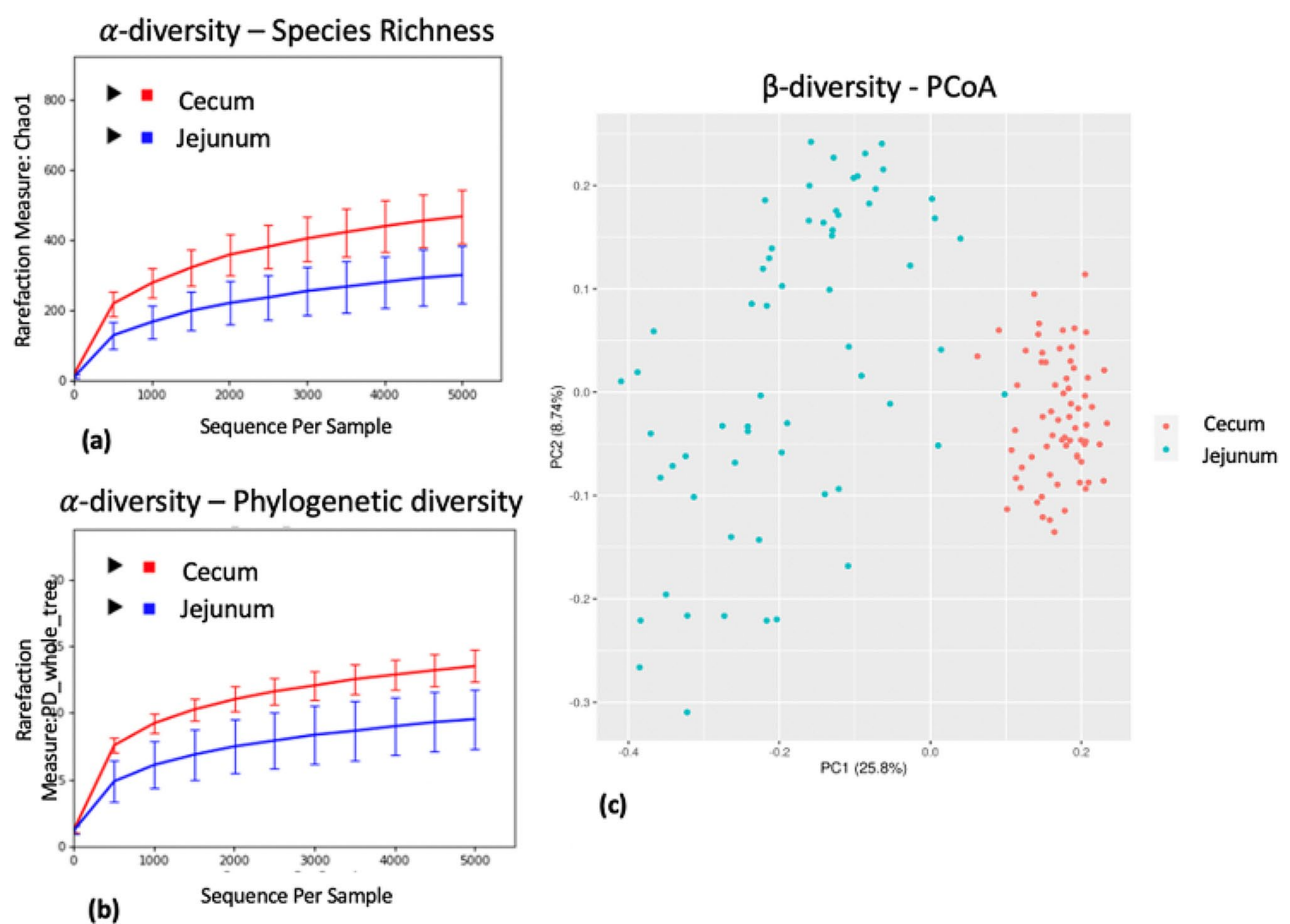
Gut site effect (jejunum vs. cecum) on microbiota diversity. ?-diversity rarefaction curves of jejunum vs. cecum indicate that there is a significant difference depending on the gut site, based on the species richness (Chao1) (**a**) and phylogenetic diversity (PD_whole_tree) (**b**). ?-diversity demonstrates, through Principal Coordinate Analysis (PCoA) of unweighted UniFrac distances, that the overall microbiota population is modulated by gut site. Two clustering are formed based on the cecum (red) and jejunum (blue) samples (**c**)

LEfSe results analysis showed that more than 100 taxa belonging to different taxonomic levels were differentially abundant between the jejunum and cecum microbiota composition (LDA?>?2). At the phylum level, multiple bacterial strains belonging to *Proteobacteria* and *Cyanobacteria* were predominant in the jejunum while the cecum was mostly inhabited by some strains of *Bacteroidetes* and *Verrucomicrobia*. At family level, jejunum showed higher abundance of *Lactobacillaceae* (LDA?>?4), *Enterococcaceae*, *Aerrococcaceae, Planococcaceae, Corynebaceriaceae* (LDA?>?2), while *Bacteroidaceae* and *Coriobacteriaceae* (LDA?>?2) were more dominant in the cecum. At genus level, the jejunum displayed a markedly higher abundance of *Lactobacillus, Enterococcus, Streptococcus and Lactococcus* (LDA?>?4) compared to the cecum while *Ruminococcus* and *Blautia* (LDA?>?4) were more abundant in the cecum. The Cladogram generated from LEfSe allows the visualization of these bacteria, from the phylum to the genus level (Fig.?7).

**Fig. 7:**
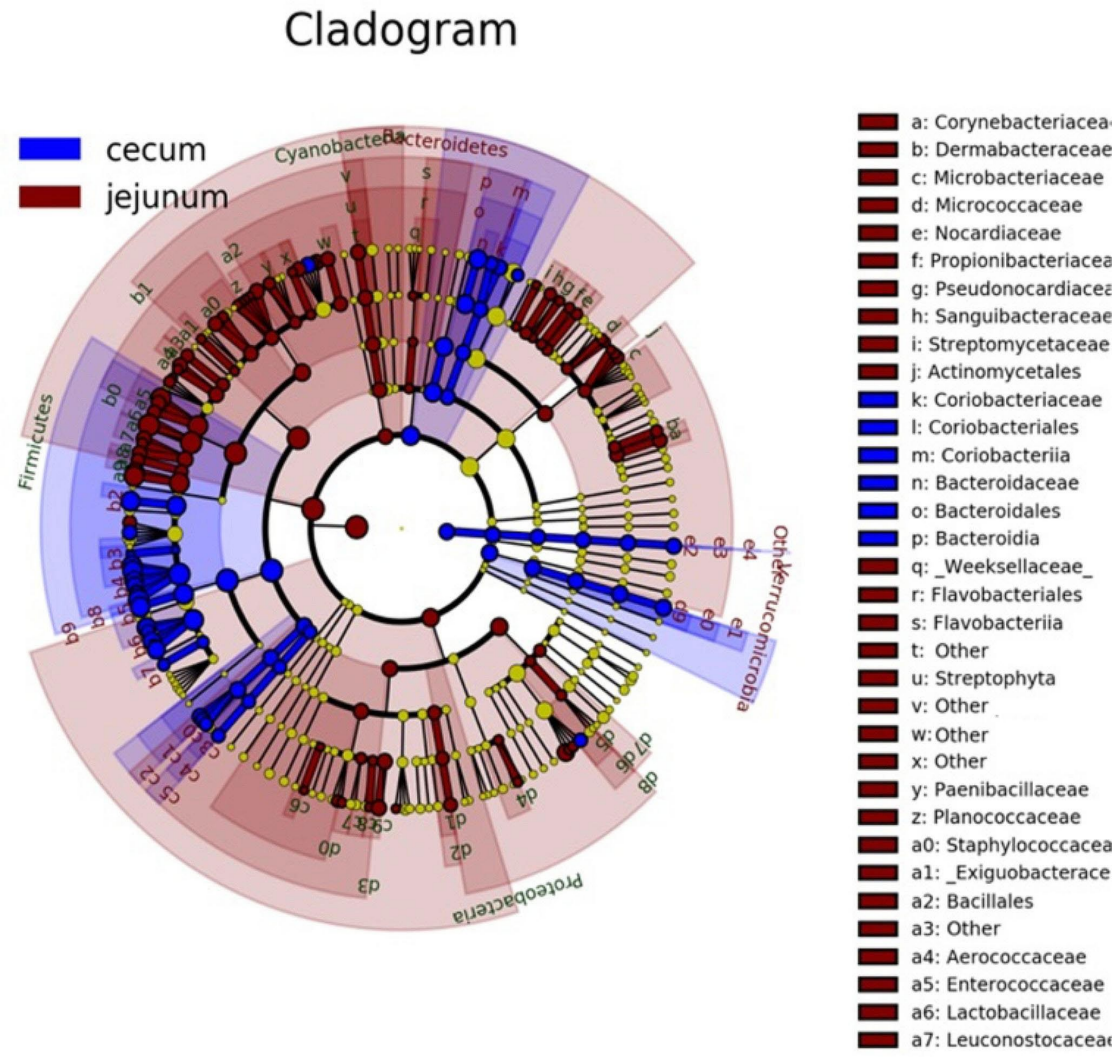
Bacterial differences between gut sites (jejunum vs. cecum). Linear discriminant analysis (LDA) effect size (LEfSe) cladogram indicates the significant bacteria from phylum (inner circle) to genus levels (outer circle), which are modulated by the two tissues (*p*?<?0.05). Biomarker taxa are heighted by shaded areas and coloured circles. The list of bacteria in brown represents the ones which are significantly modulated by jejunum while blue indicates the ones mostly modulated by the cecum site. The cut-off value of LDA was 2 or higher

## Discussion

In this study high throughput 16SrRNA gene sequencing was used to investigate the jejunum and cecum microbiota of individual broiler chickens fed a control (CTL), citrus (CTS) and cucumber (CMB) supplementary diet over a 4-week production cycle. ?-Diversity and ?-diversity analysis showed the overall bacterial microbiota composition was significantly affected by the gut site (*p*?<?0.001) but not by either of the dietary supplements, CTS and CMB, at both 14 and 28 days of age. However, the Linear discriminant analysis (LDA) effect size (LEfSE) demonstrated that the CTS diet modulated several bacterial strains in the jejunum (*Lactobacillus, Enterococcus, Clostridium*) and cecum (*Blautia, Enterococcus*) while only a bacterial strain (*Bacillus*) was influenced by the CMB diet in the cecum of broilers.

### Microbiota profile of the jejunum and cecum

In this study, the results showed that the cecum microbiota displayed a greater richness and diversity bacterial community compared to the jejunum. Overall, the observed microbial diversity was generally lower than those reported in other animals. This phenomenon has been associated with the rapid transit of food through the digestive tract of birds (Wei et al. 2013; Rougi?re and Carr? 2010). In the present work, the microbiota of the jejunum was predominantly composed of Firmicutes bacteria, and within this phylum, the majority belonged to the *Lactobacillus, Enterococcus, Streptococcus* and *Lactococcus* genus. These bacteria all belong to the *Lactobacillales* order and they represented almost 45% of the total jejunal microbial community. Similar results have been previously reported in chickens (Shang et al. 2018; Cuccato et al. 2021; Xiao et al. 2017; Stamilla et al. 2021). It has also been found that the diverse microbial communities that inhabit the jejunum are similar to those in the ileum (Gong et al. 2007). Bacteroidetes and Actinobacteria were the dominant flora of broilers cecum microbiota with the greatest abundance of *Bacteroides*. These findings are consistent with previous 16S rRNA gene- based studies conducted on chickens (Xiao et al. 2017; Huang et al. 2021; Dauksiene et al. 2021).

### Effects of dietary supplements, CTS and CMB, on the gut microbiota

Neither of the supplementary diets, CTS and CMB, used in this study showed a significant modulation of the overall jejunum or cecum microbiota. The literature suggests that the overall chicken intestinal microbiota is more likely to be modulated by gut site rather than supplementary diets (Ballou et al. 2016). The resulting effect also depends on the type of manipulation performed; in studies using ABGPs or LPS, the entire microbiota composition was found to be strongly affected (D?az et al. 2018; Pourabedin et al. 2015; Lucke et al. 2018). According to Ballou et al. 2016; dietary treatments generally stimulate a greater microbial differentiation at day 14, whereas a more stable microbial taxonomy can be detected at day 28. However, in the current study, bird age (14 and 28 days old) did not reveal a higher impact on gut microbiota and therefore, datasets at both time points were combined. Overall, a promising CTS effect on the modulation of beneficial bacterial strains has been observed in the jejunum and caecum of broilers while the CMB diet did not appear to have a significant impact. The CMB diet only influenced the *Bacillus* genus in the broiler cecum but no relevant information in terms of promising dietary effects was found associated to its bioactive compounds.

### Effects of CTS dietary supplements on the jejunal microbiota

The linear discriminant analysis (LDA) effect size (LEfSE) analysis revealed that the CTS dietary supplements significantly increased the counts of *Lactobacillus* (*p*?<?0.01) and decreased the counts of *Enterococcus* (*p*?<?0.01) and *Clostridium* (*p*?<?0.05) in the jejunum at both ages.

Several strains of *Lactobacillus* are widely used as probiotics with associated anti-inflammatory and anti-microbial activities in humans and animals (Corth?sy et al. 2007). Although their mode of action is not fully characterised, *Lactobacillus* based treatments have been shown to ameliorate the digestion, absorption of nutrients (Vieco-Saiz et al. 2019) and the fermentation of dietary fibres to produce SCFAs (Besten et al. 2013). Several studies have demonstrated the beneficial effects of SCFAs in the regulation of the poultry gut health (Liu et al. 2021a; Brisbin et al. 2011). SCFAs serve as a source of energy for the intestinal cells and exert protective effects against enteric pathogens (Mao et al. 2019). Thus, the greater abundance of *Lactobacillus* in the jejunum in the current study was interpreted as being that the CTS diet potentially having a positive effect in terms of gut function and health.

*Enterococci* are physiologically part of the gut microbiota of broilers, but they are typically opportunistic pathogens, and their function relies upon the species (Dolka et al. 2017). For example, *E. cecorum* has been found to cause infections in broilers (Jung et al. 2017). Likewise, *E. faecalis* and *E. durans* have been frequently associated with poultry diseases, especially endocarditis (Velkers et al. 2011). In France, the growing occurrence of *Enterococci* in poultry farms over the last 15 years has been related to the presence of this strain in the flock (Souillard et al. 2022). Many strains of *E. faecium* and *E. faecalis* have been found to be resistant to all currently available antibiotics (Miller et al. 2014). On the other hand, *E. faecium* is used commercially as a probiotic supplement in broiler diets where its use has been observed to increase the microbial diversity, enhance the intestinal absorbance and resistance to infections (Samli et al. 2007). Unfortunately, the 16srRNA sequencing technique cannot accurately discriminate among the bacterial species so the *Enterococcus* decreased has been interpreted as likely to be beneficial.

The genus *Clostridium* is well known in the poultry sector, mainly because of the pathogenic species *C. perfringens*. However, it is important to state that most of the *Clostridia* bacteria are non-pathogenic commensals of the gut and many are even beneficial for animals (Rinttil? and Apajalahti 2013). Indeed, many *Clostridium* species have been reported to participate in biological activities and are recognised to have a huge potential as probiotics (Guo et al. 2020). The study of Biddle et al. 2013 confirmed that some *Clostridium* bacteria can use complex plant-derived carbohydrates to produce SCFAs in broilers. In the absence of any other available data, it is suggested that the shift in abundance of *Clostridium* is linked to the observed increase in abundance of *Lactobacillus* and may be considered beneficial for the broiler?s gut health.

### Effects of CTS dietary supplements on the caecal microbiota

In the cecum, the linear discriminant analysis (LDA) effect size (LEfSE) showed that CTS diet significantly increased the counts of *Blautia* (*p*?<?0.01) while decreased the counts of *Enterococcus* (*p*?<?0.05) at both ages.

*Blautia* belongs to the genus of anaerobic bacteria with probiotic activity, commonly found in the gut and faeces of mammals. According to Kiu et al. 2019; the cecum microbiota of healthy broilers appears to have a higher abundance of the genera *Blautia*. Members of the *Blautia* genus are known to be SCFAs producers in the gut, and reductions in this genus have previously been associated with a *C. jejuni* infection model (Mountzouris et al. 2007; Yu et al. 2019). For this reason, *Blautia spp*. may act as a key beneficial microbiota member, serving to enhance intestinal health of broilers and preventing pathogenic microbes successfully colonising and initiating disease. This genus may also be implicated in roles associated with biotransformation and crosstalk with other intestinal microorganisms, as well as in the inhibition of the insulin signalling and the fat accumulation, as demonstrated in humans (Liu et al. 2021b). The decreased abundance of *Enterococci* was consistent in both the jejunum and cecum and therefore considered as likely to be beneficial.

### Interaction between the CTS bioactive compounds and the gut microbiota

The correlation between CTS bioactive compounds and modulation of microbial strains was investigated. The citrus extract?s bioactive compounds such as polyphenols, essential oils, pectin, carotenoids, or vitamins (single or interaction effect) might explain the modulation of selective bacteria in this study. This may be further complicated by gut environment modifications such as pH changes induced by some bacterial strains, which could increase, or decrease the abundance of other bacteria.

Polyphenols are recognised to possess prebiotic properties which support the growth of selective bacteria by acting as a source of nutrient supply (Mar?n et al. 2015). Among these bacteria, *Lactobacilli* and *Bifidobacteria* populations, are known to be increased by polyphenols (Iqbal et al. 2020). Flavonoids for example show antimicrobial potential against certain bacteria such as *Staphylococcus aureus, Escherichia coli* and *Campylobacter* thanks to their ability to modulate the gut microbiota of broilers. The study of Tolnai et al. 2021 identified a decrease in the abundance of *Clostridium* in birds fed with flavonoids and highlighted its association with a consequent alteration in bile biotransformation. Based on these considerations, the increase in abundance of *Lactobacilli* and decrease in abundance of *Clostridium* in the jejunum of broilers fed with the CTS diet could be associated to the CTS polyphenol content.

Essential oils, generally extracted from the peel of citrus fruits, contain high amounts of limonene and linalool which have been demonstrated to be able to inhibit pathogenic bacteria in the small intestine of chickens (Mitsch et al. 2004; Bruggeman et al. 2002). Several studies on broilers reported that essential oils can increase the number of lactic acids producing bacteria, such as *Lactobacilli*, and decreased the count of *E. coli* in the jejunum (Tiihonen et al. 2010; Erhan et al.?^2017^). The antibacterial effect of citrus peel oils could explain the observed increased in of *Lactobacilli* in the jejunum of broilers fed with the CTS diet.

Furthermore, citrus extract contains a high abundance of pectin. This dietary fibre can escape digestion and absorption, so this characteristic makes it a good candidate in the regulation of the gut microbiota (Mahmood and Guo 2020). In other species, differences in bacterial strains at the jejunum and cecum level can be influenced by pectin (Wiese 2019). However, the mechanism of action of pectin in broilers in vivo nutrition trails is not clearly defined. In vitro studies have demonstrated the ability of pectin to participate in the immune response of broilers (?vila et al. 2021) but no evidence has been demonstrated on the modulation of the microbiota.

The study of Tolnai et al. 2021 showed that carotenoids can reduce *Enterococcaceae* and *Clostridiaceae* families while positively modulating the abundances of the genus *Lactobacillus* in broilers. Despite the finding that carotenoids may potentially modulate the such bacterial strains, their mechanism of action requires further investigation.

CTS diet is also a source of vitamin C which exerts anti-inflammatory and immune- modulatory properties. Broilers have the ability to synthesize vitamin C in kidneys which, in addition to higher level of vitamin C supplemented by the diet, can alter the microbiota and contrast pathogens as *Salmonella* (Gan et al. 2020). It has also been found that vitamin C is associated with the increase of beneficial bacteria *Lactobacillus* and *Bifidobacterium* in animals (Yang et al. 2020) but little information is available in broilers. This means that based on our result, the higher abundance of *Lactobacilli* in CTS diet could be also attributed to the presence of vitamin C in the extract. Further studies would be required to clarify the mechanisms of action of these bioactive dietary compounds. Future investigations may also consider greater extract dosage and methods of extraction.

## Conclusion

This study showed that the dietary supplements, CTS and CMB, did not affect the overall microbiota composition of broilers at both 14 and 28 days of age. However, the CTS diet influenced the abundance of beneficial bacteria such as *Lactobacillus, Enterococcus* and *Clostridium* in the jejunum and *Blautia and Enterococcus* in the cecum, while the CMB diet only influenced the abundance of *Bacillus* in the cecum. Most bacteria modulated by the CTS diet are likely to have a positive impact on the gut health, in the absorption of nutrients, production of SCFAs and possibly even the stimulation of the immune system to certain challenges. However, the mechanism of action of the dietary bioactive compounds that can modulate these beneficial strains needs to be clarified. Therefore, despite the likely positive effects of CTS dietary supplement on the gut health, further investigation is required to justify its use as antimicrobial growth promoters (ABGPs) in broiler chickens.

### Supplementary Information

Below is the link to the electronic supplementary material.

Supplementary Material 1.?Growth performance data. (137 KB)

Supplementary Material 2.? Effect of bird age on the jejunal and caecal microbiota (14 and 28?day). (217 KB)

## Data Availability

The datasets, scripts and metadata that support the findings of this study have been deposited in the Figshare repository under the Microbiota investigation 10.6084/m9.figshare.24716583. (https://doi.org/10.6084/m9.figshare.24716583.v1)

## Abbreviations

ABGPs: Antibiotic growth promoters
AMR: Antimicrobial resistance
ANOVA: Analysis of variance
BW: Body weight
CMB: Cucumber diet
CTL: Control diet
CTS: Citrus diet
FI: Feed Intake
LC-MS/MS: Liquid chromatography-tandem mass spectrometry
LDA: Linear discriminant analysis
LEfSe: Linear discriminant analysis Effect Size
LPS: Lipopolysaccharide
OTU: Operational taxonomic units
PCoA: Principal Coordinate Analysis
PERMANOVA: Permutational multivariate analysis of variance
SCFAs: Short chain fatty acids
16s rRNA: 16?S ribosomal ribonucleic acid
