# Supplementary material for: Measuring the impact of dietary supplementation with citrus or cucumber extract on chicken gut microbiota using 16s rRNA gene sequencing
Source: Vet Res Commun. 2024 May 23;48(4):2369–84. doi: 10.1007/s11259-024-10417-w (PMC11315731; doi:10.1007/s11259-024-10417-w)
Supplement: Supplementary file 1 — Supplementary Material 1.?Growth performance data. (137 KB) [file 11259_2024_10417_MOESM1_ESM.pdf]

Measuring the impact of dietary supplementation with citrus or cucumber extract on chicken gut microbiota using 16s rRNA gene sequencing

Journal of Veterinary Research Communications

Francesca Riva, David *McGuinness*, Dorothy E. F. McKeegan, Jorge Peinado-Izaguerri, Geert Bruggeman, David Hermans, Peter D. Eckersall, Mark McLaughlin, Maureen Bain

Corresponding author: Dr. Mark McLaughlin (mark.mclaughlin@glasgow.ac.uk), School of Biodiversity, One Health and Veterinary Medicine, University of Glasgow, Bearsden Rd, Glasgow, G61 1QH, United Kingdom

Growth performance data

The body weight (BW) and Feed Intake (FI) of broiler chickens were recorded to evaluate the broiler's growth performance. The BW of broiler chickens was evaluated at day 0, (N=108), 14 (N=105) and 28 (N=34) using a weight balance (0.001g). The sex of each bird was determined by dissection and the females were excluded from the data. The feed intake (FI) was measured at day 0, 14 and 28 per feeder per pen. A general linear model (GLM) and Tukey test was run on RStudio (version 4.0.0) to test the main effects of diet at each bird age. A significant difference in BW was observed at day 14 and 28 ($p < 0.05$) with the birds in the CTL diet being heavier than either of the CTS or CMB diets (Table S1).

		Day 0				*Day 14*				*Day 28*			
	Diet	N	Mean	±	SE	N	Mean	±	SE	N	Mean	±	SE
BW	CTL	36	0.035	±	0.000[a]	35	0.441	±	0.007[c]	11	1.392	±	0.032[g]
	CTS	36	0.038	±	0.000[a]	35	0.379	±	0.008[d]	12	1.228	±	0.073[h]
	CMB	36	0.037	±	0.001[a]	35	0.374	±	0.007[d]	11	1.218	±	0.034[h]

Table S1 Body weight of broilers fed CTL, CTS and CMB supplementary diets at 0,14 and 28 days of age. The table indicates the number of broilers (N), the mean and standard error of the mean (SE). Means that do not share a letter are significantly different.

In terms of FI, no differences were observed comparing the CTL and CTS dietary supplements at both ages while broilers fed the CMB diet showed a significant lower FI compared to the ones under the CTL and CTS diet at 14 and 28 days old ($p < 0.05$) (Table S2).

		Day 14				*Day 28*			
	Diet	N	Mean		SE	N	Mean		SE
FI	CTL	35	0.052	±	0.001^{c}	11	0.124	±	0.002^{h}
	CTS	35	0.052	±	0.001^{c}	12	0.120	±	0.003^{h}
	CMB	35	0.048	±	0.000^{d}	11	0.110	±	0.003^{i}

Table S2 Feed Intake of broilers fed CTL, CTS and CMB supplementary diets at 0,14 and 28 days of age. The table indicates the number of pens (N), mean and standard error of the mean (SE) referred CTL, CTS and CMB diet. Means that do not share a letter are significantly different.
