# Supplementary material for: Measuring the impact of dietary supplementation with citrus or cucumber extract on chicken gut microbiota using 16s rRNA gene sequencing
Source: Vet Res Commun. 2024 May 23;48(4):2369–84. doi: 10.1007/s11259-024-10417-w (PMC11315731; doi:10.1007/s11259-024-10417-w)
Supplement: Supplementary file 2 — Supplementary Material 2.? Effect of bird age on the jejunal and caecal microbiota (14 and 28?day). (217 KB) [file 11259_2024_10417_MOESM2_ESM.pdf]

Measuring the impact of dietary supplementation with citrus or cucumber extract on chicken gut microbiota using 16s rRNA gene sequencing

Journal of Veterinary Research Communications

Francesca Riva, David *McGuinness*, Dorothy E. F. McKeegan, Jorge Peinado-Izaguerri, Geert Bruggeman, David Hermans, Peter D. Eckersall, Mark McLaughlin, Maureen Bain

Corresponding author: Dr. Mark McLaughlin (mark.mclaughlin@glasgow.ac.uk), School of Biodiversity, One Health and Veterinary Medicine, University of Glasgow, Bearsden Rd, Glasgow, G61 1QH, United Kingdom

Effect of bird age on the jejunal and caecal microbiota (day 14 and 28)

The same workflow, used to explore differences based on dietary treatments and tissue, was applied to investigate the influence of bird age on the jejunal and caecal microbiota.

In the jejunum, no significant differences were found comparing the α-diversity indexes at day 14 and 28 of age ($p > 0.05$). Rarefaction curves, generated by Chao1 and PD_whole tree, indicate good sequencing depth to cover all the OTUs. However, no difference in terms of within samples bacterial diversity was discovered based on bird age (Figure S1A and S1B). The Permutational Multivariate Analysis of Variance (PERMANOVA) has indicated that β-diversity metrics were not statistically different between day 14 and 28 ($p > 0.05$). The PCoA plot show that bird age had no significant impact on the jejunal microbiota composition (Figure S1C).

In the cecum, bird age has a slight effect only based on phylogenetic diversity (PD whole tree index) ($p < 0.05$) but not species richness (Chao1 index) (Figure S2A, S2B). Permutational Multivariate Analysis of Variance (PERMANOVA) tests showed that β-diversity metrics between the two groups was statistically different ($p < 0.05$). In the unweighted PCoA plot,

samples show two clustering based on bird age (Figure S2C). However, due to the slight effect of bird age only on the cecum, the analyses of dietary effects were performed combining datasets at a tissue-specific level to maximize data utilization.

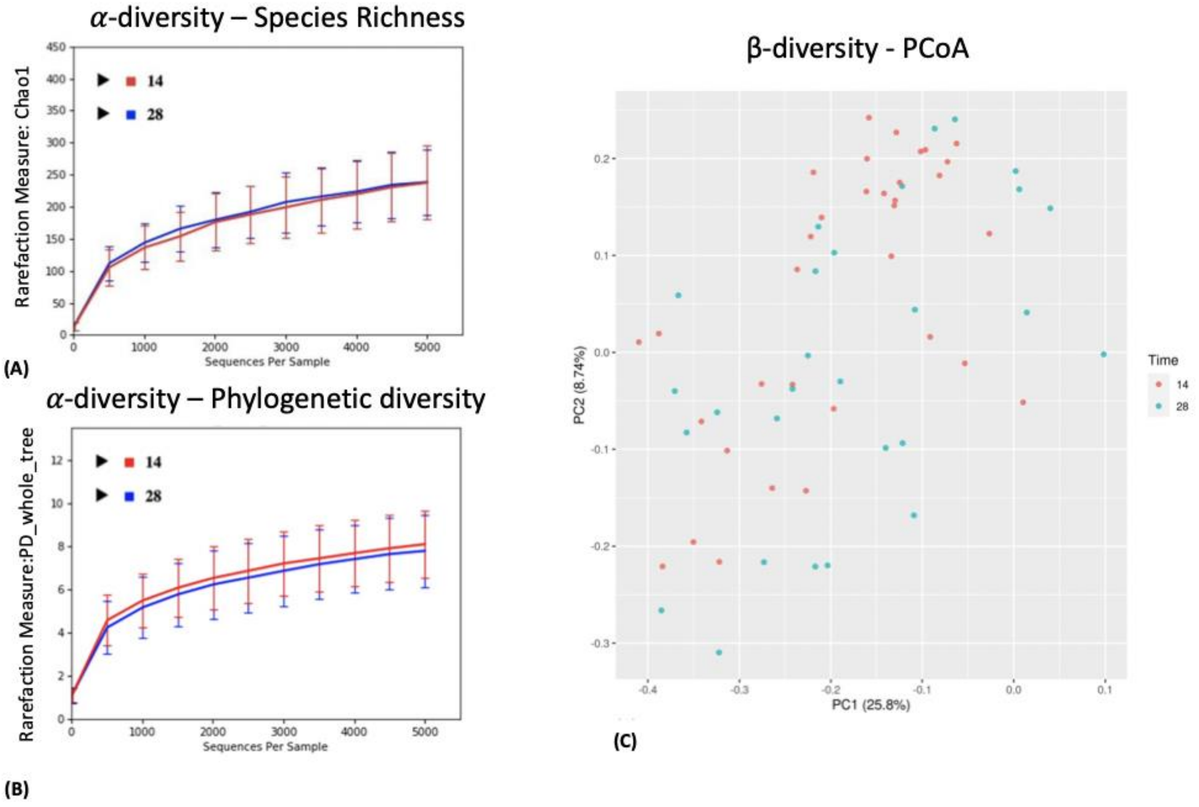

Fig S1 Bird age effect on diversity in the jejunal microbiota
α -diversity rarefaction curves of samples at 14 and 28d of bird age are investigated with both Chao1 **(A)** and PD_whole_tree **(B)** indexes. No within samples microbial differences are identified based on bird's age. β-diversity, through Principal component analysis (PCoA) of unweighted UniFrac distances, does not show clustering based on bird age **(C)**. Bird age therefore has no impact on the overall microbiota composition of the jejunum.

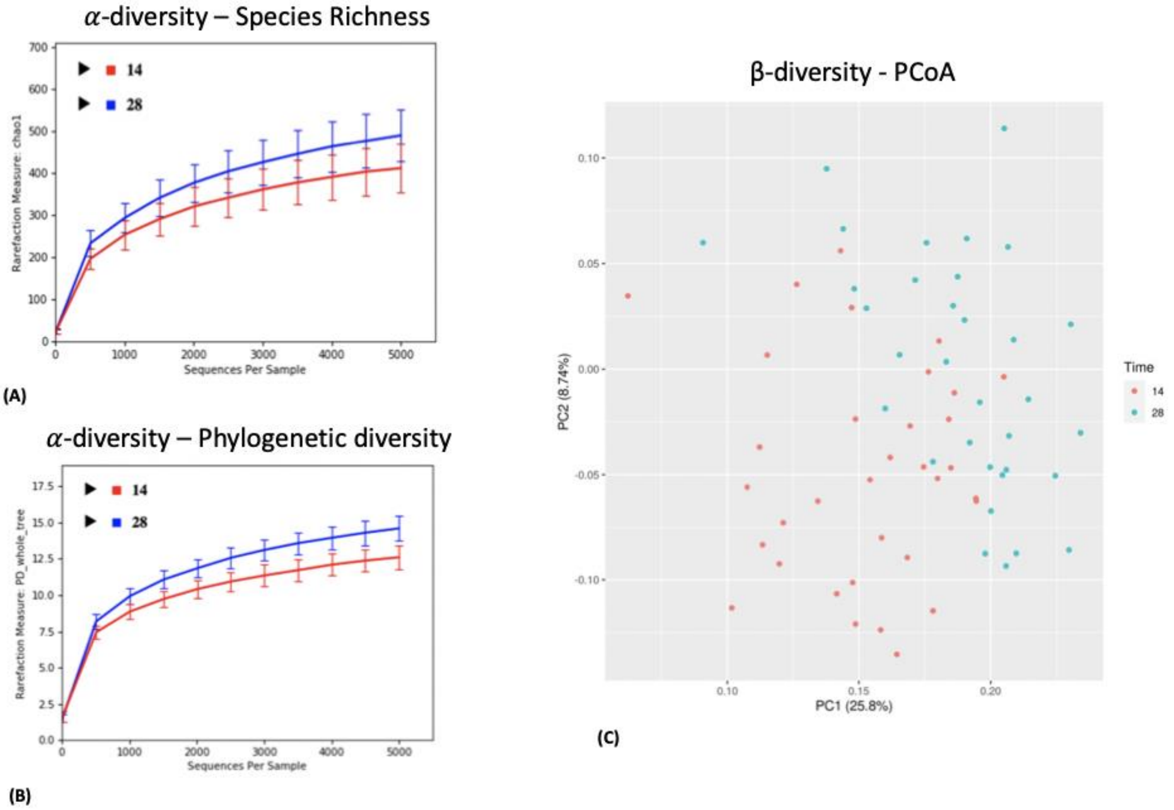

Fig S2 Bird age effect on diversity in the caecal microbiota

α-diversity rarefaction curves of broilers at 14 and 28 days of cecum samples were investigated with both Chao1 **(A)** and PD whole tree **(B)** indexes. A significant difference ($p < 0.05$) can be observed just in term of phylogenetic diversity but not richness. Principal component analysis (PCoA) plot of β-diversity analysis shows clustering based on bird age **(C).** Bird age therefore has a slight effect on the overall microbial community of the cecum.
